# Solitary plasmacytoma of thoracic vertebra in a woman with Lynch syndrome: A case report

**DOI:** 10.1016/j.ijscr.2019.10.052

**Published:** 2019-10-28

**Authors:** E.F. Röpke, F. Theissig, G. Ulrich, K. Bäker, C. Bochwitz, A. Grundig, C. Paasch

**Affiliations:** a Department of Orthopaedics, Traumatology and Spine Surgery, Helios Klinik Jerichower Land, August - Bebel - Strasse 55a, 39288, Burg, Germany; b Institute of Pathology, Helios Klinikum Emil von Behring, Walterhöferstrasse 11, 14165, Berlin, Germany; c Radiologie Sudenburg, Halberstädter Str. 125 - 127, 39112, Magdeburg, Germany; d Department of General, Visceral and Cancer Surgery, Helios Klinikum Berlin-Buch, Schwanebecker Chaussee 50, 13125, Berlin, Germany

**Keywords:** Solitary plasmacytoma, Lynch syndrome, Spinal tumor, DNA mismatch repair, Case report

## Abstract

**Introduction:**

One of the major disabling health conditions among elderly is back pain due to degenerative diseases. Less than 1% of spine disorders are caused by malignancies. Among the rare primary vertebral neoplasms the multiple myeloma and the plasmacytoma account for 26% of these cases.

**Presentation of case:**

We are reporting a case of 64 year-old woman, who suffered from progressive upper back pain and intermittent neurological symptoms including lower limb weakness and voiding disorder under axial loading. Her medical history includes a Lynch syndrome (LS). Computed tomography (CT) and magnetic resonance imaging (MRI) detected a single malignant osteolytic process of the spine involving T5. Urgent surgery with laminectomy intralesional tumor removal and posterior stabilization (Th4–Th6) due to unstable pathologic fracture with spinal cord compression was conducted after interdisciplinary decision. Histopathological examination of the tumor revealed a solitary plasmacytoma.

**Discussion:**

To our knowledge this is the first case report of a solitary plasmacytoma of the bone (SPB) that arise in a patient who suffers from LS. Similar DNA mismatch repair malfunction is existent in LS and SPB. Hence, a hereditary correlation might be imaginable.

**Conclusion:**

When detecting a lytic spinal tumor in a patient who suffers from LS a SPB should be taken under consideration.

## Highlights

• Similar oncogenesis in solitary plasmacytoma and Lynch syndrome assumed.
• The unknown metachronous malignancy in a Lynch syndrome patient.
• Don’t sit and wait with elderly and enduring back pain.

## 1 Introduction

One of the major disabling health conditions among elderly is back pain caused by degenerative diseases [1,2]. It is crucial not to miss the less than 1% of malignant disorders of the spine [3]. The majority of malignant spinal tumors are metastases [4]. Less than 10% are primary tumors of the vertebral column [5]. Among these primary vertebral malignancies the multiple myeloma (MM) and the plasmacytoma make up to 26%. These neoplasmas base on a monoclonal plasma cell proliferation. They appear as a single lesion (solitary plasmacytoma) or as a multiple lesion (MM), producing a monoclonal immunoglobulin. In terms of their location the solitary plasmacytoma can be differentiated into the SPB and the solitary extramedullary plasmacytoma (SEP). The incidence of SPB is approximately 40% higher than SEP. The median age at diagnosis is 55–60 years. Male are more often affected than women [2:1] [6]. A familial predisposition is known but the pathway of inheritance has not been revealed yet. In the majority of cases the vertebral bodies of the thoracic spine are involved by SPB. Radiological findings are vertebral body osteolysis with pathologic fracture and soft tissue masses with consecutive spinal cord compression [5]. Treatment of choice is symptom control with local radiation, surgical procedures, if necessary, and oncologic aftercare to prevent the turnover to multiple myeloma [7].

We are reporting a case of a 64 year-old woman who suffered from a LS and a SPB involving thoracic vertebra 5. This work has been reported in line with the SCARE criteria [8].

## 2 Presentation of case

**Fig. 1:**
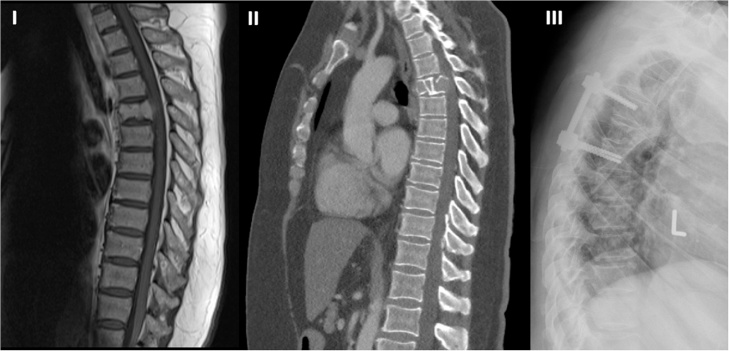
I) Preoperative sagittal T1 weighted magnetic resonance imaging showing a hypointense lesion with dorsal extrusion in T5 and less than 50% vertebral body collapse. II) Sagittal computed tomography scan showing expansile irregular osteolytic lesion of T5 vertebral body and involvement of the anterior and posterior wall. III) Lateral thoracic radiography after decompression of T5 and dorsal instrumented stabilization of T4–T6.

**Fig. 2:**
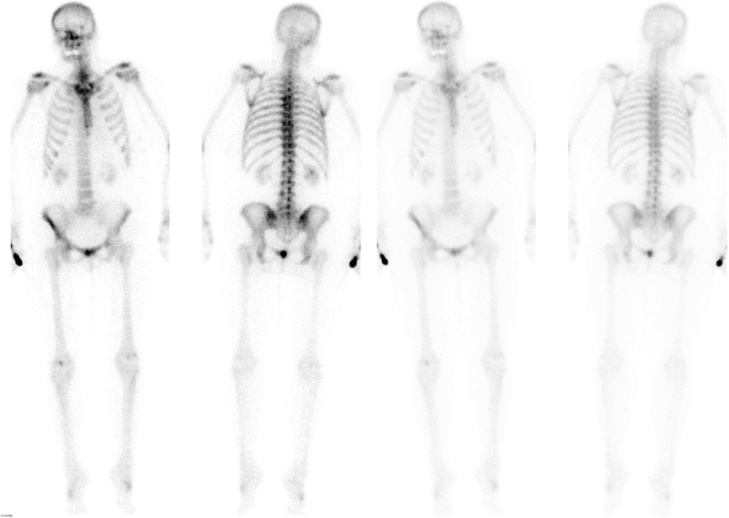
Skeletal scintigraphy with 690MBq Tc-99m-DPD did not reveal any malignant or metastatic process.

A 64-year-old female presented with progressive back pain at our hospital. Previously 6 month of outpatient conservative treatment led to no recovery of the symptoms. On time of admission in the emergency room she suffered from worsening upper back pain and intermittent neurological symptoms including lower limb weakness and voiding disorder under axial loading. The patient reported that cancer surgery of rectum, colon and uterus due to LS had taken place 10 years ago. Sporadic oncological aftercare was conducted the past 5 years. The additional medical history includes a first degree relative who suffers from LS. Neither patient’s vital signs and blood tests nor urine analyses revealed any inflammatory processes. Weight loss, fever and night sweat were negated. CT and MRI detected a single malignant osteolytic process of the spine involving T5 with a pathologic fracture leading to segmental kyphosis (Fig. 1). Epidural soft tissue masses with typical curtain sign were causing spinal chord compression [9]. Skeletal scintigraphy (Fig. 2) and single photon emission computed tomography (SPECT) could not match the MRI findings. The CT scan did not reveal other primary malignant or metastatic processes.

Differential diagnosis, causing patient’s symptoms, like osteoporotic fracture with posterior wall displacement, myelopathy, spondylodiscitis and other primary vertebral tumors were next to metastatic malignancies interdisciplinary discussed.

**Fig. 3:**
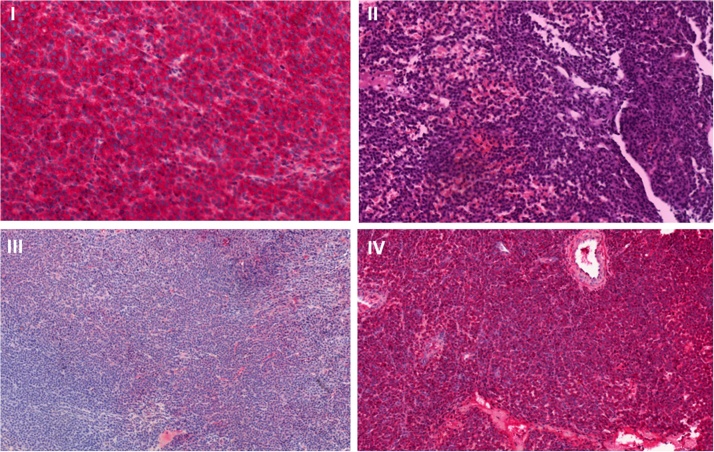
Resected tumor tissue specimen. I) CD138 positively stained specimen, marking a plasmacytic derivation (×40). II) Masses of plasma cells (H&E, ×40). III) Immunhistological study stained negatively with anti lambda - light chain antibodies (×40). IV) Immunhistological study stained positively with anti kappa - light chain antibodies (×40).

The clinical symptoms increased rapidly. Hence, urgent surgery with laminectomy intralesional tumor removal and posterior stabilization (Th4-Th6) due to unstable pathologic fracture (SINS 13) with spinal cord compression was conducted after interdisciplinary decision with radiologist, oncologist and spine surgeon [10,11]. The postoperative course was uneventful. On discharge at 6th day after surgery self-suffiency and full axial loading was reached. Histopathologic findings revealed a plasma cell neoplasia type kappa (Fig. 3). Iliac crest puncture did not reveal a systemic infiltration. Serum electrophoresis could not detect an M-Spike, lambda was normal, but a monoclonal gammopathy with gradient at free kappa light chains was found.

In conclusion the diagnosis of SPB was made. After case presentation at our interdisciplinary tumor board the decision for radiotherapy (RT) with 46 Gy and bisphonsphonate therapy was made. The patient is self-sufficient and occasionally depending on oral non-opioid painkiller 18 months after surgery. Neurologic symptoms vanished. A follow up CT showed a steady segmental kyphosis and a clear epidural space.

## 3 Discussion

Due to rapid neurologic symptoms and loss of spinal stability an urgent surgical therapy in the reported case was necessary from our point of view. A final preoperative determination of the dignity was not possible, which at the best wouldn’t require the surgical treatment but only single local radiation [12].

The procedure of dorsal decompression (T5 laminectomy) and instrumented stabilization of T4-T6 (EXPEDIUM 5.5 Spine System, DePuy Synthes Spine, 700 Orthopaedic Drive Warsaw, IN 46582) was done after interdisciplinary decision and in advice of the spinal instability neoplastic score of 13 [11]. The national low back pain guideline recommends symptomatic therapy (e.g. painkillers, physiotherapy) in case of non-existing red flags as first line treatment [13]. The initial treating physician was not aware of the patient’s oncologic medical history to request prompt imaging.

Plain radiography, MRI and CT are standard imaging techniques in detecting spinal disorders. More often than primary malignancies are metastatic lesions of the axial skeleton [5]. In the case report at hand we completed the diagnostic imaging with bone scintigraphy and SPECT imaging to prevent missing any metastatic process. No tracer enhancement could be found in the suspect MRI area. Retrospectively most likely it was caused by very low tracer uptake due to slow metabolic activity of the SPB [14].

Our patient also suffered from LS. It is a dominantly inherited cancer predisposition syndrome and the most common cause of inherited colorectal cancer (2–4%) [15]. To our knowledge, this is the first case report of a SPB that arise in a patient who suffers from LS, after reviewing the literature using Google Scholar and Pubmed. Extracolonic manifestations of the LS are rectal cancer, ovarian, endometrial, gastric, urothelial/renal, brain, pancraeticobillary cancer as well as skin and small bowel malignancies [16,17]. Interestingly also hematopoietic malignancies like lymphoma, myeloma and leukemia may arise in patients who are suffering from LS. These tumor entities are associated with a MSH2 mutation like other extracolonic manifestations of LS. MSH2 is a DNA mismatch repair protein, encoded by the MSH2 gene on chromosome 2. Summarized this DNA mismatch repair malfunction is existent in LS and plasma cell proliferative diseases like SPB [18,19]. Hence, a hereditary correlation might be imaginable. We assumed that due to earlier death of LS patients in the” pre genetic analysis days” a diagnostic and a correlation lack of these tumors might be possible.

After histopathological results and exclusion of a plasma cell myeloma oncologic treatment following the SPB therapy recommendations were initiated. Local RT was applied at the tumor site. Nevertheless according to literature and guidelines on this topic there seems to be no consensus about the optimal radiation dose [20,21]. The recommendation to conduct RT bases on retrospective studies. Randomized clinical trials are needed to reveal more evidence. Patients with SPB and LS should stay in continuous oncologic aftercare due to 49.9% incidence of progression to MM [7].

## 4 Conclusion

Back pain of the elder is a common everyday symptom. Early diagnostic imaging has to be considered in medical history of cancer. When detecting a lytic spinal tumor in a patient who suffers from LS a SPB should be taken under consideration.

To our knowledge, this is the first case report of a SPB that arise in a patient who suffers from LS. A DNA mismatch repair malfunction, as cancer pathogenesis, is existent in LS and SPB. Hence, a hereditary correlation might be possible.

## Sources of funding

This research did not receive any specific grant from funding agencies in the public, commercial, or not-for-profit sectors.

## Ethical approval

This study was not applicable for ethical approval.

## Consent

I have obtained written consent for publication of this case report from the patient and I can provide this should the Editor ask to see it.

## Author’s contribution

Dr. med. Ekkehard Friedrich Röpke (corresponding author): Contribution to the paper: first author, data collection, data analysis and interpretation, writing the paper.

PD Dr. med. Franz Theissig: Contribution to the paper: histopathological examination of the tumor, interpretation of the histological pictures.

Dr. med. Carsten Bochwitz: Contribution to the paper: treatment and examination of the patient.

Dr. med. Katharina Bäker: Contribution to the paper: treatment and examination of the patient.

Dr.med. Alexander Grundig: Contribution to the paper: treatment and examination of the patient.

Dr. med. Gerhard Ulrich: Contribution to the paper: bone scintigraphy examination and interpretation.

Dr. med. Christoph Paasch: Contribution to the paper: data analysis and interpretation, supervising writing of the paper.

## Registration of research studies

The case report at hand is not a first-in-man case report of a novel technology or surgical technique, therefore a registration of these case reports according to Declaration of Helsinki 2013 is not required.

## Guarantor

Dr. med. Ekkehard Friedrich Röpke.

## Provenance and peer review

Commissioned, externally peer-reviewed.

## Declaration of Competing Interest

None.
